# Antibodies against *M. Bovis* 65 KDa Heat Shock Protein and Its P180-188 Epitope in Sera of Patients with Juvenile Idiopathic Arthritis

**Published:** 2007-09

**Authors:** Denisa Zlacka, Jiri Velek, Pavla Vavrincova, Ilona Hromadnikova

**Affiliations:** 1 *Department of Molecular Biology and Cell Pathology, Clinic of Obstetrics and Gynecology, 3^rd^ Medical Faculty, Charles University, Prague, Czech Republic;*; 2 *Department of Biological Chemistry Institute of Organic Chemistry and Biochemistry, Academy of Sciences of the Czech Republic, Prague, Czech Republic;*; 3 *Rheumatology Outpatient Department, University Hospital Motol, 2^nd^ Medical Faculty, Charles University, Prague, Czech Republic*

**Keywords:** enzyme-linked immunosorbent assay, epitope, heat shock protein, juvenile idiopathic arthritis

## Abstract

We screened the levels of antibodies to *M. bovis* hsp65 and the 180-188 epitope by using ELISA in a cohort of 72 juvenile idiopathic arthritis (JIA) patients and 38 healthy controls. We analysed an association between antibody levels and rheumatoid factor, antinuclear antibodies, human leukocyte antigen B27 and the severity and the duration of the disease. The majority of anti-hsp65 antibodies in a cohort of JIA patients were of the IgG isotype (54.2%) with IgM (13.9%) antibodies increased to a lesser degree. IgG antibodies to *M. bovis* hsp65 (*P*<0.001) and the 180-188 epitope (*P*<0.001) were significantly increased in all of three disease onset types when compared with healthy controls. The highest levels of IgG antibodies to *M. bovis* hsp65 and its P180-188 epitope were observed in oligoarthritis and in patients with no X-ray changes and functional limitation, while the lowest antibody levels were detected in patients with the most severe stage of articular damage. When antibody levels to *M. bovis* hsp65 and the 180-188 epitope were examined within patient and control populations, significantly higher levels of IgG and IgM antibodies to *M. bovis* hsp65 were observed in both JIA (*P*<0.001) and healthy control (*P*<0.001) cohorts. These findings may suggest that the high levels of IgG antibodies to *M. bovis* hsp65 and its P180-188 epitope would reflect the least serious cases of JIA. Since IgM antibodies to *M. bovis* hsp65 and P180-188 *M. bovis* hsp65 epitope exceeded the control level in a few patients with JIA we believe they are not of concern.

## INTRODUCTION

Current evidence suggests that heat shock proteins (hsps) may be important elements in the infectious aetiology and pathogenesis of various autoimmune diseases involving rheumatoid and juvenile idiopathic arthritis (1-4).

The hsps are produced by prokaryotic and eukaryotic cells in response to heat and to a variety of the other stress inducing agents (5, 6). They are highly evolutionarily conserved and behave as dominant immunogens known to induce very strong humoral and cellular immune responses in numerous infections caused by bacteria, protozoa, fungi and nematodes, as well as in various experimental infection models (7-10).

Recently, we even reported that patients who had developed a severe infection or septicaemia caused by *Klebsiella pneumoniae* and *Aspergillus fumigatus* during earlier or later post-transplant time periods had augmented levels of total Ig, IgG and IgM isotype antibodies to *M. bovis* hsp65 despite ongoing treatment with immunosuppressive drugs (11).

Humoral immune responses to hsps have been observed in a number of autoimmune diseases. Increased levels of anti-hsp65 antibodies in serum have been found in ankylosing spondylitis (12), RA (12, 13), psoriasis (14), systemic sclerosis (15), primary Raynaud’s phenomenon (15) and Kawasaki disease (16).

Moreover, strong correlation between anti-hsp 65 antibodies and carotid atherosclerosis was observed (17, 18).

Cross-reactivity to self hsps may play a role in autoimmune diseases to some extent. Cross-reactive autoantigens in certain circumstances may incite an autoimmune response that contributes to the initiation of autoimmune inflammation. Molecular mimicry is considered as an explanation i.e. for the pathogenesis of primary biliary cirrhosis since antibodies to mycobacterial hsp65 cross-react with main mitochondrial antigens (19).

The mycobacterial 65 kDa heat shock protein (Hsp65) is of critical significance in the experimental model of adjuvant arthritis (AA), which is an extensively studied form of experimental arthritis with a close histopathologic resemblance to rheumatoid arthritis (RA) and juvenile idiopathic arthritis (JIA) (20-22). The disease can be induced in susceptible rats by immunization with incomplete Freund's adjuvant containing heat-killed mycobacteria (23) or by passive transfer of a single T cell clone recognising the 180 to 188 amino acid sequence in *M. bovis* hsp65 (21). This T cell clone was also shown to be reactive with cartilage proteoglycan, aggrecan (24). The proteoglycans of articular cartilage consist of core proteins of variable length with different oligosaccharide and glycosaminoglycan chains linked to them. The molecular weight of aggrecan in the tissue ranges from 1 to 3.5 MDa, and their size based on electron microscopy varies in the range of 100-300 nm. They form large aggregates (molecular size up to 800 MDa) with hyaluronan and so-called link-protein. The percentage of aggregating proteoglycans is 50-85% of total weight of proteoglycans. Self cross-reactivity or mimicry of T cells based on the sequence homology between the 180-188 *M. bovis* hsp65 sequence and cartilage proteoglycan might explain the ability to induce autoimmune inflammation. Vice versa, pre-immunization with mycobacterial hsp65 protects against induction of AA (23, 25). The resistance to AA can be transferred to susceptible rats as well as by intravenous (i.v.) infusion of antibodies against hsp65 derived from resistant strains (26).

Juvenile idiopathic arthritis is an autoimmune disease in which autoreactive T cells play a central role in joint inflammation and destruction (27). JIA encompasses a heterogeneous group of rheumatic diseases with three principal types of onset involving oligoarthritis, polyarthritis and systemic disease which can be distinguished on the basis of clinical features presenting during the first 3 month of the disease (28, 29). The aetiology of JIA is still unknown and the pathogenesis mechanism remains unclear.

Just a few previous studies examined humoral reactivity to *M. bovis* hsp65 and the 180-188 sequence of hsp65 in patients with JIA. Ó Nualláin, *et al*. (30) reported raised levels of IgM and IgG anti-hsp65 antibodies in all subgroups of JCA (juvenile chronic arthritis) by using an enzyme-linked immunosorbent assay (ELISA). Danieli, *et al*. (31) have found antibodies to mycobacterial hsp65 of the IgG isotype in all types of JCA patients with active disease and observed somewhat greater antibody reactivity to the 180-188 peptide than to the whole hsp65 molecule by using a standard solid phase radioimmunoassay (RIA).

The current study was designed to screen and compare the levels of antibodies to *M. bovis* hsp65 and *M. bovis* hsp65 derived P180-188 synthetic peptide by using ELISA in a cohort of patients with JIA. We analysed also an association between anti-hsp antibody levels and rheumatoid factor (RF), antinuclear antibodies (ANA), HLA B27 (human leukocyte antigen B27), disease duration (less than 2 years versus more than 2 years) and the severity of the disease evaluated on the base of Steinbrocker’s functional classification and rtg (X-ray, Roentgen ray) staging system.

## MATERIALS AND METHODS

### Patients

72 JIA patients (32 males, 40 females) aged 3-56 years (mean 16.5, median 13) from the Outpatient Department of Rheumatology, University Hospital Motol in Prague and 38 age-matched healthy controls (aged 2-47 years, mean 17.2, median 15) were included in the study.

Three patients at the age of 46, 50 and 56 years were included in the study. All of them fulfilled diagnostic criteria for JIA since the disease onset occurred before their 16^th^ birthday.

The underlying diseases, using the Idiopathic Arthritis of Childhood Classification criteria (28) were 22 oligoarthritis, 34 polyarthritis (12 RF positive and 22 RF negative) and 16 systemic diseases. A total cohort involved 14 RF positive and 58 RF negative; 14 ANA positive and 58 ANA negative; 11 HLA B27 positive and 54 HLA B27 negative patients (in 7 patients HLA B27 was not determined).

The Steinbrocker’s functional classification was used to rate the extent of physical disability on a four-level scale, ranging from stage I to stage IV (32). The distribution of the patients according to the Steinbrocker’s functional classification, was as follows: 33 patients (46.5%) stage I, 28 patients (39.4%) stage II and 10 patients (14.1%) stage III. Rtg changes were graded with the classical Steinbrocker’s Staging System (I-IV). No rtg changes were found in 27 patients (39.7%). Rtg changes of stage I were detected in 13 patients (19.1%), stage II in 12 patients (17.6%), stage III in 11 patients (16.2%) and stage IV in 5 patients (7.4%).

We tested 13 patients with early (0-2 years) and 54 patients with established disease (patients with duration of the disease over 2 years).

Patients were treated depending on the stage of the disease with non-steroid antirheumatics (NSAIDs), corticosteroids and/or disease modifying antirheumatics (DMARDs). None of the JIA patients as well as non-autoimmune controls had suffered a recent infection.

Enzyme immunoassays (EIA RF IgA, EIA RF IgG, EIA RF IgM, Test-line, Clinical Diagnostics, Czech Republic) were used for the detection of IgA, IgG and IgM rheumatoid factor in patients´ serum or plasma samples.

Sera were stored at -80°C until used. Local ethics committee approval and informed consent were obtained for all individuals involved in this study.

### Synthesis and purification of *M. bovis* hsp65 derived peptide (180-188)

The 180-188 peptide derived from *M. bovis* hsp65 (TFGLQLELT) was synthesized manually by solid phase method on 4-methylbenzhydrylamine resin using Boc/Bzl strategy, which was described previously (33). Three-fold molar excess of protected amino acid was used in the coupling step. Dicyclohexylcarbodiimid/1-hydroxybenzotriazole activation was used. After synthesis the peptide-resin was washed with DMF, methyl *terc*-butylether and dried. The detachment of the peptide from the resin and the side chains deprotection were carried out by liquid hydrogen fluoride-anisole (9:1). The obtained peptide was purified by reverse phase high performance liquid chromatography (RP-HPLC). The peptide sequence was assigned by amino acid analysis and fast atom bombardment mass spectrometry (FAB-MS) spectra. Lyophilised peptides were entirely soluble in deionised sterile water.

### Measurement of antibodies to *M. bovis* hsp65 and 180-188 peptide by ELISA

Reactivity to *M. bovis* hsp65 and its derived peptide was examined by using ELISA. 96-well ELISA plates (Peptide coating kit, Takara, Japan) were coated with 50 μl of synthetic peptide and/or *M. bovis* hsp65 (Lionex, Braunschweig, Germany) at concentration 0.2 μg/well for 2 h at room temperature. After washing in distilled deionised water, plates were blocked in blocking buffer (Takara) for 1 h at room temperature. Then 100 μl of serum samples diluted 1:100 in PBST/1% BSA (PBS, pH 7.4 containing 0.05% Tween 20, Sigma Biosciences, St Louis, MO, USA) were added to the wells in duplicates and incubated overnight at 4°C. After washing in PBST, plates were incubated with secondary peroxidase conjugated antibodies (Sigma Biosciences, St Louis, MO, USA) for 5 h at the room temperature. To determine the levels of IgM and IgG antibodies to *M. bovis* hsp65 and 180-188 epitope goat-anti-human IgM (μ-chain specific, Sigma Biosciences, St Louis, MO, USA) diluted 1:50 000 and goat-anti-human IgG (Fab fragment specific, Sigma Biosciences, St Louis, MO, USA) diluted 1:40 000 were employed. The plates were then washed in PBST and developed with 0.4 mg/ml of o-phenylenediamine dichloride (OPD), 0.4 mg/ml urea hydrogen peroxide in 0.05 M phosphate citrate buffer, pH 5.0 (Sigma FAST OPD, Sigma Biosciences, St Louis, MO, USA) and incubated in dark at the room temperature for 30 minutes. Optical density was measured at 450 nm using ELISA plate reader (Dynex Technologies, MRX II, USA).

The assay for anti-hsp65 was developed by using mouse anti- *M. bovis* hsp65 monoclonal antibody (SPA-882, Stressgen, Canada) and anti-hsp highly positive sera from patients who developed severe infection or septicaemia. Patient’s serum samples were considered to be positive if the OD values exceeded the mean plus 2 SD of healthy control sera.

### Statistical analysis

For the comparison of antibody levels to *M. bovis* hsp65 and 180-188 epitope between two groups, the two-tailed Student’s t-test and nonparametric Mann-Whitney test were applied (SPSS, Statistical Package for the Social Sciences, SPSS Inc., Illinois, USA). Two-tailed t-test has been usually recommended to obviate the situation that the significance test would have a greater power.

*P* values of less than 0.05 were regarded as significant. The results of statistical analysis were consistent in most cases.

## RESULTS

We compared the levels of IgG and IgM isotype antibodies against *M. bovis* hsp65 and synthetic peptide 180-188 derived from *M. bovis* hsp65 between patients with JIA and healthy controls. The background of the assay (OD values obtained from the wells in which patient’s sera were substituted with PBST/1% BSA) was less than 0.05 OD unit.

*M. bovis* hsp65 reacted in all cases with anti- *M. bovis* hsp65 monoclonal antibody (SPA-882). The absorbance in these wells was more than 1.00 OD unit, however monoclonal antibody did not react with a peptide-derived sequence of *M. bovis* hsp65. Sera of patients with severe acute infection depending on aetiological agents (*Klebsiella pneumoniae, Pseudomonas aeruginosa, Aspergillus fumigatus*) reacted with *M. bovis* hsp65 as well as *M. bovis* hsp65 derived synthetic peptide.

### Antibodies to *M. bovis* hsp65

**Figure 1:**
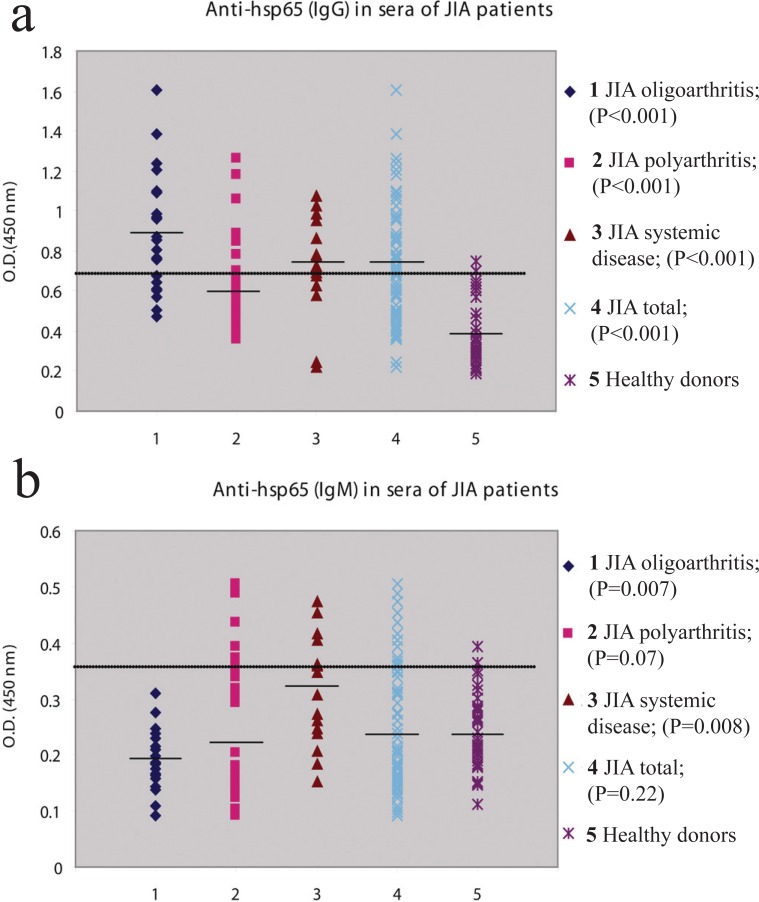
(a), IgG antibodies to *M. bovis* hsp65 in patients with various subgroups of JIA and healthy individuals. Results are expressed as OD values. The horizontal line indicates the upper limit calculated as 2 SD above mean OD of the healthy controls; (b), IgM antibodies to *M. bovis* hsp65 in patients with various subgroups of JIA and healthy individuals. Results are expressed as OD values. The horizontal line indicates the upper limit calculated as 2 SD above mean OD of the healthy controls.

We screened the levels of IgM and IgG antibodies to *M. bovis* hsp65 and 180-188 epitope in a cohort of 72 JIA patients involving 22 oligoarthritis, 34 polyarthritis (RF positive and RF negative) and 16 systemic arthritis. IgG antibodies to *M. bovis* hsp65 exceeded the upper limits of the normal in a group of 54.2% (39/72) JIA patients and 5.3% (2/38) healthy individuals (Figure 1a, b). IgG antibodies to *M. bovis* hsp65 were detected in all JIA subgroups (16/22, 72.7% oligoarthritis; 11/34, 32.3% polyarthritis and 12/16, 75% systemic disease). IgM anti-hsp65 antibodies were elevated in 13.9% (10/72) of JIA patients and 2.6% (1/38) of healthy controls. IgM positivity was detected only in polyarticular (5/34, 14.7%) and systemic disease (5/16, 31.2%) groups.

JIA sera showed significantly increased levels of IgG antibodies to *M. bovis* hsp65 in all three disease onset types (*P*<0.001) when compared with healthy controls. Significantly elevated levels of IgM antibodies to *M. bovis* hsp65 were found in the systemic disease group (*P*=0.008). Elevated levels of IgM anti-hsp65 antibodies were also observed in polyarthritis, where the autoimmune response reached nearly statistical significance (*P*=0.07). Oligoarthritis produced significantly lower levels of IgM anti-hsp65 antibodies when compared with healthy controls (*P*=0.007).

### Antibodies to the 180-188 epitope

**Figure 2:**
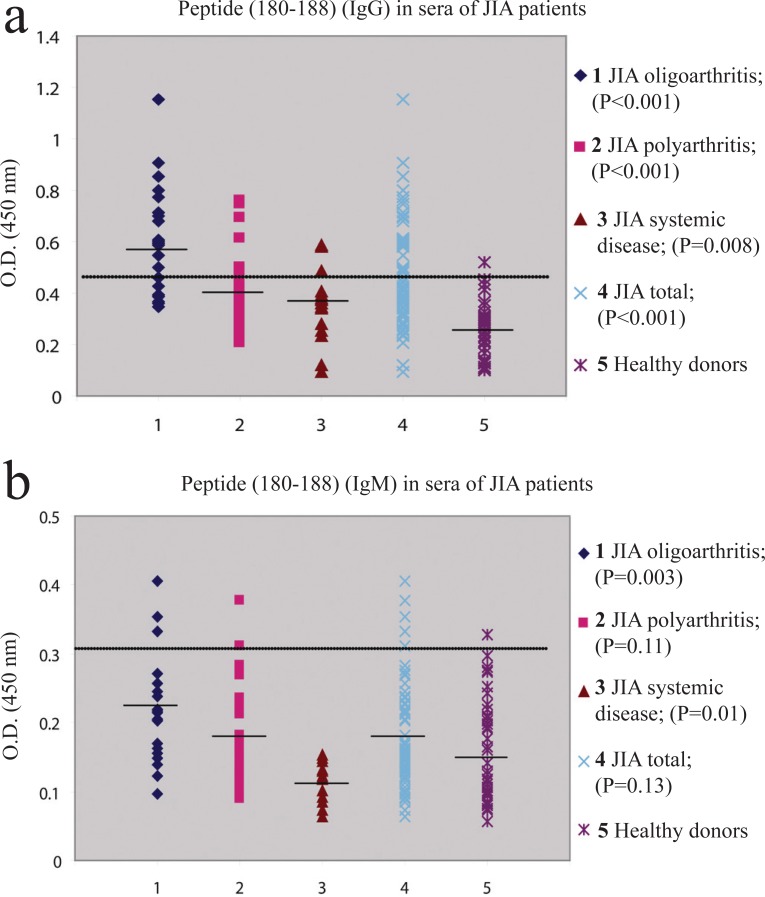
(a), IgG antibodies to 180-188 sequence of *M. bovis* hsp65 (TFGLQLELT) in patients with various subgroups of JIA and healthy individuals. Results are expressed as OD values. The horizontal line indicates the upper limit calculated as 2 SD above mean OD of the healthy controls; (b), IgM antibodies to 180-188 sequence of *M. bovis* hsp65 (TFGLQLELT) in patients with various subgroups of JIA and healthy individuals. Results are expressed as OD values. The horizontal line indicates the upper limit calculated as 2 SD above mean OD of the healthy controls.

IgG antibodies to the 180-188 epitope were detected in 2.6% (1/38) age-matched healthy individuals and 38.9% (28/72) of JIA patients involving 68.2% (15/22) oligoarthritis; 26.5% (9/34) polyarthritis and 25% (4/16) systemic disease. IgM antibodies occurred in 2.6% (1/38) of healthy controls and 8.3% (6/72) JIA patients involving 18.2% (4/22) oligoarthritis and 5.9% (2/34) polyarthritis (Figure 2a, b).

Similarly, significantly raised levels of IgG antibodies to the 180-188 sequence of *M. bovis* hsp65 were observed in each disease group comparing to control cohort, however only oligoarthritis (*P*=0.003) was found to have significantly elevated levels of IgM isotype. Systemic disease produced significantly lower levels of IgM antibodies to the 180-188 sequence of *M. bovis* hsp65 when compared with healthy controls (*P*=0.01).

### Comparison of antibody levels to *M. bovis* hsp65 and the 180-188 sequence of *M. bovis* hsp65 within patient and control cohorts

**Figure 3:**
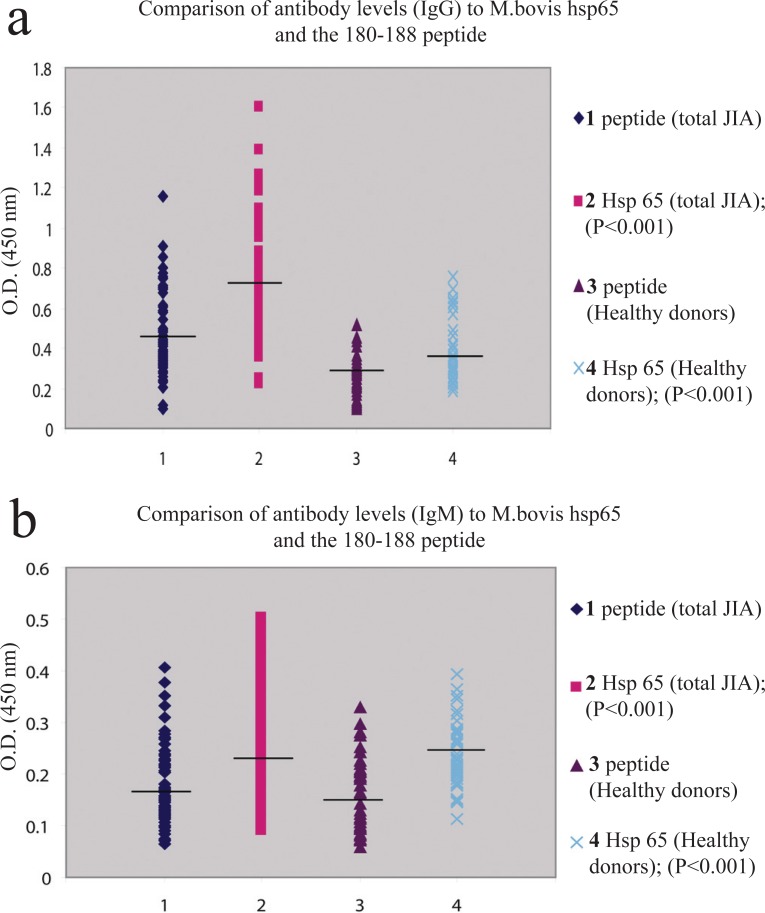
(a), Comparison of IgG antibodies to *M. bovis* hsp65 and 180-188 sequence of *M. bovis* hsp65 within JIA and control cohorts. Results are expressed as OD values. (b), Comparison of IgM antibodies to *M. bovis* hsp65 and 180-188 sequence of *M. bovis* hsp65 within JIA and control cohorts. Results are expressed as OD values.

Comparison of antibody levels to *M. bovis* hsp65 and the 180-188 sequence of *M. bovis* hsp65 were examined within patient and control populations (Figure 3a, b). Significantly higher levels of IgG and IgM antibodies to *M. bovis* hsp65 were observed in both JIA (*P*<0.001) and healthy control (*P*<0.001) cohorts.

### Influence of clinical characteristics on antibody response to *M. bovis* hsp65 and the 180-188 sequence of *M. bovis* hsp65

No association between IgG and IgM antibody levels to *M. bovis* hsp65 and the presence of ANA, HLA B27 and disease duration (0-2 years versus 3-45 years) in total JIA cohort was observed (Table 1a). However, significantly higher anti-hsp65 antibody levels of either IgG (*P*=0.008) and IgM (*P*<0.001) isotypes were found in RF positive JIA patients. No correlation was observed between antibody levels to the 180-188 sequence of *M. bovis* hsp65 and the presence of RF, ANA and HLA B27 and disease duration.

**Table 1a:** Influence of clinical characteristics on antibody response to *M. bovis* hsp65 and the 180-188 sequence of *M. bovis* hsp65

total JIA cohort (Cut-off=mean+2SD of healthy controls)	RF pos. (14)	ANA pos. (14)	HLA-B27 pos. (11)	disease duration 0-2 (13)
	versus	versus	versus	vesrus
	RF neg. (58)	ANA neg. (58)	HLA-B27 neg. (54)	3-45 (54) years

	OD mean values			
	*P* values (Student’s t test)			
	*P* values (Mann-Whitney test)			

**anti-hsp65 (IgG)** (0.672)	0.86 vs. 0.69	0.74 vs.0.71	0.60 vs. 0.76	0.70 vs. 0.74
	*P*=0.02	*P*=0.42	*P*=0.10	*P*=0.30
	*P*=0.008	*P*=0.66	*P*=0.15	*P*=0.43
**anti-hsp65 (IgM)** (0.368)	0.37 vs. 0.21	0.25 vs. 0.23	0.21 vs. 0.25	0.21 vs. 0.25
	*P*<0.001	*P*=0.29	*P*=0.13	*P*=0.12
	*P*<0.001	*P*=0.75	*P*=0.20	*P*=0.25
**180-188 epitope (IgG)** (0.455)	0.47 vs. 0.45	0.46 vs. 0.45	0.39 vs. 0.47	0.49 vs. 0.45
	*P*=0.37	*P*=0.40	*P*=0.10	*P*=0.24
	*P*=0.28	*P*=0.92	*P*=0.18	*P*=0.49
**180-188 epitope (IgM)** (0.308)	0.15 vs. 0.19	0.18 vs. 0.18	0.18 vs. 0.18	0.18 vs. 0.18
	*P*=0.17	*P*=0.38	*P*=0.48	*P*=0.48
	*P*=0.12	*P*=0.58	*P*=0.50	*P*=0.81

The declining levels of IgG anti-hsp65 and anti-hsp65 P180-188 epitope antibodies reflected the severity of the disease evaluated on the base of Steinbrocker’s functional classification and rtg staging system (Table 1b, 1c). The lowest levels of IgG anti-hsp65 and anti-hsp65 P180-188 epitope antibodies were found in JIA patients with the most severe rtg changes and physical disability.

**Table 1b:** An association between antibodies to *M. bovis* hsp65 and the 180-188 epitope and the severity of the disease evaluated on the base of Steinbrocker’s functional classification

total JIA cohort (Cut-off=mean+2SD of healthy controls) No.	Steinbrocker’s functional classification					
	OD mean values					
	stage I (33)	stage II (28)	stage III (10)	*P* values		
				Student’s t test		Mann-Whitney test

**anti-hsp65**	0.78	0.72	0.53	I vs. III	<0.001	0.014
**(IgG)**				I vs. II+III	0.056	0.25
(0.672)				II vs.vIII	0.003	0.024
**anti-hsp65**	0.22	0.27	0.21	I vs. III	0.420	0.77
**(IgM)**				I vs. II+III	0.060	0.20
(0.368)				II vs. III	0.070	0.12
**180-188 epitope**	0.51	0.43	0.32	I vs. III	<0.001	0.005
**(IgG)**				I vs. II + III	0.010	0.05
(0.455)				II vs. III	0.003	0.02
**180-188 epitope**	0.19	0.17	0.15	I vs. III	0.080	0.18
**(IgM)**				I vs. II + III	0.080	0.19
(0.308)				II vs. III	0.240	0.42

**Table 1c:** An association between antibodies to *M. bovis* hsp65 and the 180-188 epitope and the severity of the disease evaluated on the base of Steinbrocker’s rtg staging system

total JIA cohort (Cut-off=mean+2SD of healthy controls) No.	Steinbrocker’s rtg staging system						
	OD mean values						
	stage 0 (27)	stage I (13)	stage II (12)	stage III+IV (16)	*P* values		
					Student’s t test		Mann- Whitney test

**anti-hsp65 (IgG)** (0.672)	0.82	0.62	0.70	0.60	0 vs. III + IV	0.01	0.03
					0 vs. I	0.03	0.03
					0 vs. I + II + III + IV	0.007	0.02
					I vs. II + III + IV	0.36	0.53
					II vs. III + IV	0.10	0.20
**anti-hsp65 (IgM)** (0.368)	0.23	0.20	0.27	0.26	0 vs. III + IV	0.24	0.95
					0 vs. I	0.14	0.11
					0 vs. I + II + III + IV	0.34	0.68
					I vs. II + III + IV	0.05	0.16
					II vs. III + IV	0.44	0.57
**180-188 epitope (IgG)** (0.455)	0.52	0.41	0.44	0.36	0 vs. III + IV	0.002	0.02
					0 vs. I	0.06	0.14
					0 vs. I + II + III + IV	0.008	0.03
					I vs. II + III + IV	0.35	0.96
					II vs. III + IV	0.06	0.17
**180-188 epitope (IgM)** (0.308)	0.20	0.21	0.18	0.15	0 vs. III + IV	0.009	0.05
					0 vs. I	0.32	0.67
					0 vs. I + II + III + IV	0.18	0.31
					I vs. II + III + IV	0.04	0.14
					II vs. III + IV	0.13	0.63

The lowest levels of IgG anti-hsp65 and anti-hsp65 P180-188 epitope antibodies were found in JIA patients with the most severe rtg changes.

No association between IgM anti-hsp65 and anti-hsp65 P180-188 epitope antibodies and the severity of the disease was observed (Table 1b, 1c).

## DISCUSSION

In this study, we investigated the presence of antibodies to *M. bovis* hsp65 and the 180-188 peptide derived from *M. bovis* hsp65 in sera of patients with JIA and age-matched healthy individuals. The majority of anti-hsp65 antibodies in a cohort of JIA patients were of the IgG isotype (54.2%) with IgM (13.9%) antibodies increased to a lesser degree. JIA sera showed significantly increased levels of IgG antibodies to *M. bovis* hsp65 in all three disease onset types. Significantly elevated levels of IgM antibodies to *M. bovis* hsp65 were found in the systemic disease. Oligoathritis group lacked to produce IgM antibodies to *M. bovis* hsp65. These data are partially consistent with those reported by Ó Nualláin, *et al*. (30) who observed increased levels of IgM and IgG anti-hsp65 antibodies in all JCA subgroups.

Similarly, significantly raised levels of IgG antibodies to the 180-188 epitope were observed in each disease group, however only oligoarthritis was found to have significantly elevated levels of IgM isotype. Patients with systemic disease showed low antibody response to the 180-188 peptide. Our data confirmed the observation of Danieli, *et al*. (31) who reported as well as the presence of the IgG antibodies to the 180-188 epitope in all JCA subcategories. However, we did not find the greater reactivity to the P180-188 peptide when compared with the whole *M. bovis* hsp65.

The present study showed also increased humoral response to *M. bovis* hsp65 in RF positive JIA patients. However, we observed no association between antibody levels to the 180-188 epitope of *M. bovis* hsp65 and the presence of RF.

We found no correlation between antibody levels to *M. bovis* hsp65 and the 180-188 sequence of *M. bovis* hsp65 and the presence of ANA, HLA B27 and disease duration. Similarly Ó Nualláin, *et al*. (30) found no correlation between antibody levels to mycobacterial hsp65 and disease duration.

The highest levels of IgG antibodies to *M. bovis* hsp65 and its P180-188 epitope were observed in oligoarthritis and in patients with no rtg changes and functional limitation, while the lowest antibody levels were detected in patients with the most severe stage of articular damage which may be caused by a diminished and/or down regulated reactivity of immune cells and antibodies in prolonged or end-stage arthritis. Several radiographic scoring methods have been developed to evaluate radiographic changes in RA. The most commonly used methods are those devised by Sharp, Larsen and van der Heijde/Sharp, and their variants (34). However, no radiographic assessment method has been achieved universal acceptance. In spite of the so-called Steinbrocker’s method is not sensitive to clinically meaningful differences, is still widely used in clinical practise (32, 35, 36) including our Rheumatology outpatient department.

Concerning IgM antibodies to *M. bovis* hsp65, the highest levels were detected in systemic disease and polyarthritis groups. Vice versa, the highest levels of IgM antibodies to P180-188 *M. bovis* hsp65 epitope were observed in oligoarthritis when compared with healthy controls. However in both cases, neither the levels of antibodies to *M. bovis* hsp65 or to P180-188 *M. bovis* hsp65 epitope were associated with the severity of the disease evaluated on the base of Steinbrocker’s functional classification and rtg staging system.

Since IgM antibodies to *M. bovis* hsp65 and P180-188 *M. bovis* hsp65 epitope exceeded the control level in a few patients with JIA we believe they are not of concern.

One of the limitations of our study is the generalization to the whole JIA population since the participants involved in the research were especially children under the age of 16 with milder disease severity and disability levels. It is possible that more disease severity items would have been elicited if we would have a chance to collect a larger sample of older adult patients who had the onset of JIA during their childhood.

An additional potential limitation is that we did not design the study to find any association between antibodies to *M. bovis* hsp65 and/or to P180-188 *M. bovis* hsp65 epitope and clinical response to active treatment evaluated using the ACR Pediatric 30 criteria (37), which have been endorsed by both the ACR and the Food and Drug Administration as the gold standard. The patients involved in the study were sampled successively apart from when the therapy was started and/or changed. The course of JIA is variable and disease activity changes with time (over periods of a few hours to weeks) and with treatment (38).

Patients with JIA may follow a monocyclic course with complete remission, a polycyclic course characterized by recurrent episodes of disease activity, or a course of persistent polyarthritis (39, 40). Concerning EULAR criteria developed to distinguish between high and low disease activity (41-44) in adult RA entirely DAS (Disease Activity Score) but not DAS28 reached concordance with ACR Pediatric 30 when used in both children and young adults with JIA (45). However DAS is not routinely used in the assessment of the disease activity in our unit.

In a summary, these findings may suggest that the high levels of IgG antibodies to *M. bovis* hsp65 and its P180-188 epitope would reflect the least serious cases of JIA. However, IgM antibodies to *M. bovis* hsp65 and P180-188 *M. bovis* hsp65 epitope do not play a role in JIA pathogenesis at all.
